# An Extended Kalman Filter-Based Attitude Tracking Algorithm for Star Sensors

**DOI:** 10.3390/s17081921

**Published:** 2017-08-21

**Authors:** Jian Li, Xinguo Wei, Guangjun Zhang

**Affiliations:** School of Instrument Science and Opto-electronics Engineering, Beijing University of Aeronautics and Astronautics, Beijing 100191, China; lijian_0355@buaa.edu.cn (J.L.); gjzhang@buaa.edu.cn (G.Z.)

**Keywords:** star sensor, attitude tracking, extended Kalman filter

## Abstract

Efficiency and reliability are key issues when a star sensor operates in tracking mode. In the case of high attitude dynamics, the performance of existing attitude tracking algorithms degenerates rapidly. In this paper an extended Kalman filtering-based attitude tracking algorithm is presented. The star sensor is modeled as a nonlinear stochastic system with the state estimate providing the three degree-of-freedom attitude quaternion and angular velocity. The star positions in the star image are predicted and measured to estimate the optimal attitude. Furthermore, all the cataloged stars observed in the sensor field-of-view according the predicted image motion are accessed using a catalog partition table to speed up the tracking, called star mapping. Software simulation and night-sky experiment are performed to validate the efficiency and reliability of the proposed method.

## 1. Introduction

Star sensors are widely used for attitude determination in both orbiting and interplanetary spacecraft [1]. A star sensor typically operates in two modes: initial acquisition and tracking mode. The difference between them is whether the approximate attitude is known. In initial acquisition without a-priori attitude input, stars inside the whole field of view (FOV) are acquired and mapped to the matching stars in the sensor’s star catalog by a search over the whole celestial sphere. The lost-in-space algorithms are well summarized by Benjamin [2]. In the case sufficient stars have been identified, the star sensor starts the calculation of attitude and goes into tracking mode. In contrast to initial acquisition mode, the sensor performs an attitude tracking and not a star tracking in tracking mode. The current attitude and angular rate is used to determine an expected attitude for the following measurement frame. The sensor uses this expected attitude and onboard star catalog to determine the star track windows to get the stars to be measured and evaluated. This method shortens the time to find the measurement stars and will also to eliminate spurious objects. Tracking mode is more efficient and reliable than initial acquisition mode and is the key operation mode in the lifetime of a star sensor. Attitude Tracking Algorithm directly affects the star sensor’s performance.

At present, a few papers are published for tracking. Li [3] and Jiang [4] used the locations of recognized stars (reference stars) in previous star image as the centers of star track windows. If there is only one observed star in the window, the star is matched with the reference star corresponding to the track window. The radius of the window is in proportion to star sensor’s angular rate. The larger the radius is, the more stars will be observed in one track window. In that case the angular distance between the observed stars and reference stars are used to find the correct match which is complicated and time consuming. In order to speed up the accessing the cataloged stars to identify the stars which enter or exit the sensor FOV, Jiang assigned the cataloged stars into sub-catalog. A partition table including all sub-catalogs is generated which is memory-consuming. Finally, according to stars transformation between inertial-based coordinate system and image plane, the star sensor’s attitude is calculated by QUEST algorithm [5].

Samaan [1] presented two new recursive methods for identifying the stars within the FOV during tracking. The spherical polygon approach is used to sort the star position vector components in *x*, *y* and *z*-axes and then to access the cataloged stars within the FOV. However, it needs angular velocity obtained from the rate gyro and runs slower than jiang’s method [4]. The star neighborhood approach is also used as a second method for star identification. In this method Samaan accesses all cataloged stars in the union of the neighborhoods of stars identified in the previous frame. Like Jiang [4], there comes the need for more memory to save the “star neighbor table”. The attitude estimator adopted in both the proposed algorithms is the “second Estimator of the Optimal Quaternion” [6].

To improve the tracking performance under high dynamic condition, Sun [7] proposed a star tracking method which combines the spot-target-based optical flow analysis and Kalman filter to determine the angular velocity. Effective positions and size of the region of interest for detecting the star spot are provided. However, the algorithm is not suitable for use in orbit as it requires a large amount of calculation. Yu [8] proposed a multiexposure imaging based star tracking method for intensified star trackers which is not applicable to traditional star trackers without intensified image detector due to the low star sensitivity. 

Typically, the key issues in tracking mode are determining the star locations within the FOV and an optimal attitude rapidly. A novel attitude tracking method is presented herein. Two techniques are introduced to improve the efficiency and reliability. Firstly, the star sensor is modeled as a nonlinear stochastic system with the state estimate providing the three degree-of-freedom attitude quaternion and angular velocity. The stochastic model uses the extended Kalman filter (EKF) [9] to estimate the attitude for the current and following frame. Secondly, inspired by [1,4], a partition table is established and accessed by spherical polygon approach involving a tradeoff between time versus memory consuming. The new tracking algorithm, presented here, is supported by both the software simulation and the hardware test results. 

The remainder of this paper is organized as follows: Section 2 describes the algorithm, including the tracking model, attitude estimation, star catalog partition and initial angular velocity estimation criteria, in detail. Section 3 presents the simulation and experiment results to demonstrate the performance of the proposed algorithm. Finally, some conclusions are drawn.

## 2. Algorithm Description

In this section, we initially describe the tracking model of the star sensor. We then provide some implementation details, particularly on attitude estimation, star catalog partition and initial angular velocity estimation. Firstly, the EKF algorithm for attitude estimation is designed. Secondly, a novel star catalog partition method is presented to speed up accessing the cataloged stars while having a small memory requirement. Thirdly, the initial angular velocity estimation algorithm is described.

### 2.1. Tracking Models

**Figure 1:**
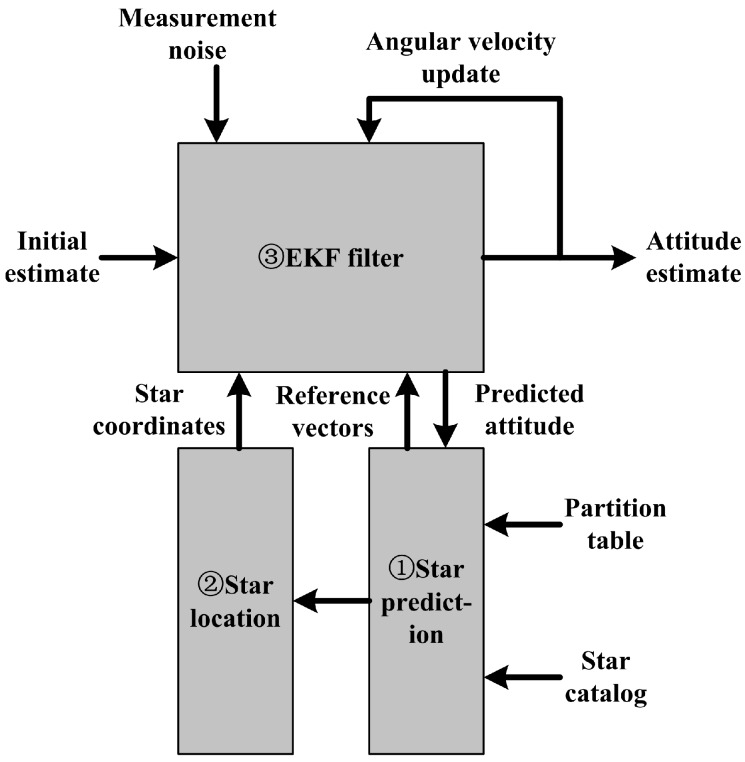
Tracking model of a star sensor.

After a full-sky star identification is finished to establish an initial attitude and angular velocity, the star sensor enters the tracking mode and recursively performs the following three steps as shown in Figure 1.

① From EKF the attitude quaternion and its error covariance is extrapolated to the next frame in EKF. The expected attitude is used to access onboard partition table and star catalog by spherical polygon approach [1] to determine the centers of the star track windows. Meanwhile, the error covariance is used to determine the radius of the track windows.
② Get the stars to be measured [10] and evaluated in the track windows of star image. If there is only one star in a reference star’s neighborhood, the star in star image is matched with the star in reference star image. Further, the match can be checked by the inter-star angles between the reference stars and the observed stars.
③ The coordinates of the matched stars in the observation star image are used as the measurements for EKF to estimate the optimal attitude quaternion of current frame and the angular velocity.

### 2.2. EKF Based Attitude Estimation

The quaternion $q=\left[\begin{array}{cccc} q_{0} & q_{1} & q_{2} & q_{3} \end{array}\right]$ and the angular velocity $w={\left[\begin{array}{ccc} w_{x} & w_{y} & w_{z} \end{array}\right]}^{T}$ are selected as state variables:

$$s={\left[\begin{array}{ccc} q_{0} & q_{1} & \begin{array}{ccc} q_{2} & q_{3} & \begin{array}{ccc} w_{x} & w_{y} & w_{z} \end{array} \end{array} \end{array}\right]}^{T}$$

The quaternion q representing a rotation is given by [11]

(1) $$q=\left[\begin{array}{c} q_{0}\\ q_{1}\\ q_{2}\\ q_{3} \end{array}\right]=\left[\begin{array}{c} \cos\frac{\theta }{2}\\ \left(\sin\frac{\theta }{2}\right)u \end{array}\right]$$

where u is the axis of rotation and θ is the angle of rotation about u. The quaternion satisfies a single constraint, which is ‖q‖=1.

A system model, that is, a function ϕ and additive noise σ required to calculate the next state in terms of the present state can be defined as [9]:

(2) $$s_{k}=\phi_{k-1}\left(s_{k-1}\right)+\sigma_{k-1}$$

σ is assumed to be additive Gaussian noise with zero mean and covariance matrix Q. The state transition function ϕ extrapolates from the state at time interval k−1 to the next state at time interval k. The angular velocities are assumed constant so that $w_{i}(t_{k})=w_{i}(t_{k-1})$.

The quaternion propagation in time must be derived. The relation between q and the angular velocity w is known to be [11]:

(3) $$\dot{q}=\frac{1}{2}\left[\begin{array}{c} 0\\ w_{x}\\ w_{y}\\ w_{z} \end{array}\right]⊗q\equiv \frac{1}{2}\Omega ⊗q\equiv \frac{1}{2}\left(\begin{array}{cc} \begin{array}{cc} 0 & -w_{x}\\ w_{x} & 0 \end{array} & \begin{array}{cc} -w_{y} & -w_{z}\\ -w_{z} & w_{y} \end{array}\\ \begin{array}{cc} w_{y} & w_{z}\\ w_{z} & -w_{y} \end{array} & \begin{array}{cc} 0 & -w_{x}\\ w_{x} & 0 \end{array} \end{array}\right)q\equiv \bar{\Omega }q$$

where the symbol ‘⊗’ represents quaternion multiplication.

Solving for q when w is constant gives:

(4) $$q\left(t\right)=e^{\bar{\Omega }\left(t-t_{k}\right)}q\left(t_{k}\right)$$

Since $\frac{2\bar{\Omega }}{\left\|\Omega \right\|}$ is an orthogonal and skew-symmetric matrix, its eigenvalues are ±i,±i [12]. After solving for the closed form of the matrix exponential [13], the solution, when simplified, is:

(5) $$q\left(t\right)=\left[\cos\left(\frac{\left\|\Omega \right\|\left(t-t_{k}\right)}{2}\right)I+\frac{2}{\left\|\Omega \right\|}\sin\left(\frac{\left\|\Omega \right\|\left(t-t_{k}\right)}{2}\right)\bar{\Omega }\right]q\left(t_{k}\right)$$

The state transitions for each variable have now been defined. The state transition function is:

(6) $$\phi_{k}(s)=\left[\begin{array}{c} Q_{\mathrm{tran}}q(t_{k})\\ w_{x}\\ w_{y}\\ w_{z} \end{array}\right]$$

where:

(7) $$Q_{\mathrm{tran}}=[\cos\left(\frac{\left\|\Omega \right\|\Delta t}{2}\right)I+\frac{2}{\left\|\Omega \right\|}\sin\left(\frac{\left\|\Omega \right\|\Delta t}{2}\right)\bar{\Omega }]$$

$Q_{\mathrm{tran}}$ is the 4-by-4 quaternion transition matrix.

The linearized state transition matrix $\Phi_{k}$ is computed as the partial derivative of the state transition function with respect to each state variable and evaluated at time $t=t_{k}$. The time dependency requires that it be computed at each state update. Formally, $\Phi_{k}$ is a matrix of dimension 7-by-7:

(8) $$\Phi_{k}=\left[\begin{array}{cc} \begin{array}{c} Q_{\mathrm{tran}}\\ \begin{array}{ccc} 0 & 0 & \begin{array}{cc} 0 & 0 \end{array}\\ 0 & 0 & \begin{array}{cc} 0 & 0 \end{array}\\ 0 & 0 & \begin{array}{cc} 0 & 0 \end{array} \end{array} \end{array} & \begin{array}{c} Q_{\sigma }\\ \begin{array}{ccc} 1 & 0 & 0\\ 0 & 1 & 0\\ 0 & 0 & 1 \end{array} \end{array} \end{array}\right]$$

where $Q_{\mathrm{tran}}$ is defined above in Equation (7). $Q_{\sigma }$ is defined as:

(9) $$Q_{\sigma }={\frac{\partial q}{\partial w}|}_{w=w_{k}}={\frac{\partial Q_{\mathrm{tran}}q(t_{k})}{\partial w}|}_{w=w_{k}}={\left[\frac{\partial Q_{\mathrm{tran}}q(t_{k})}{\partial w_{x}}\;\frac{\partial Q_{\mathrm{tran}}q(t_{k})}{\partial w_{y}}\;\frac{\partial Q_{\mathrm{tran}}q(t_{k})}{\partial w_{z}}\right]}_{w=w_{k}}$$

Each column may be computed separately as given below. The partial derivative of $Q_{\mathrm{tran}}q(t_{k})$ with respect to each $w_{i}$ is:

(10) $$\frac{\partial Q_{\mathrm{tran}}q(t_{k})}{\partial w_{i}}=\left(\frac{-w_{i}\tau }{2\left|w\right|}\sin\left(\frac{\left|w\right|\tau }{2}\right)I+\left(\frac{w_{i}\tau }{{\left|w\right|}^{2}}\cos\left(\frac{\left|w\right|\tau }{2}\right)-\frac{2w_{i}}{{\left|w\right|}^{3}}\sin\left(\frac{\left|w\right|\tau }{2}\right)\right)\bar{\Omega }+\frac{2}{\left|w\right|}\sin\left(\frac{\left|w\right|\tau }{2}\right)\frac{\partial \bar{\Omega }}{\partial w_{i}}\right)q\left(t_{k}\right)$$

The partial derivatives of $\bar{\Omega }$ with respect to $w_{x}$, $w_{y}$ and $w_{z}$ are:

(11) $$\frac{\partial \bar{\Omega }}{\partial w_{x}}=\frac{1}{2}\left[\begin{array}{cc} \begin{array}{cc} 0 & -1\\ 1 & 0 \end{array} & \begin{array}{cc} 0 & 0\\ 0 & 0 \end{array}\\ \begin{array}{cc} 0 & 0\\ 0 & 0 \end{array} & \begin{array}{cc} 0 & -1\\ 1 & 0 \end{array} \end{array}\right],\frac{\partial \bar{\Omega }}{\partial w_{y}}=\frac{1}{2}\left[\begin{array}{cc} \begin{array}{cc} 0 & 0\\ 0 & 0 \end{array} & \begin{array}{cc} -1 & 0\\ 0 & 1 \end{array}\\ \begin{array}{cc} 1 & 0\\ 0 & -1 \end{array} & \begin{array}{cc} 0 & 0\\ 0 & 0 \end{array} \end{array}\right],\frac{\partial \bar{\Omega }}{\partial w_{z}}=\frac{1}{2}\left[\begin{array}{cc} \begin{array}{cc} 0 & 0\\ 0 & 0 \end{array} & \begin{array}{cc} 0 & -1\\ -1 & 0 \end{array}\\ \begin{array}{cc} 0 & 1\\ 1 & 0 \end{array} & \begin{array}{cc} 0 & 0\\ 0 & 0 \end{array} \end{array}\right]$$

The measurement update equation is [9]:

(12) $$z_{k}=h_{k}\left(q_{k}\right)+v_{k}$$

**Figure 2:**
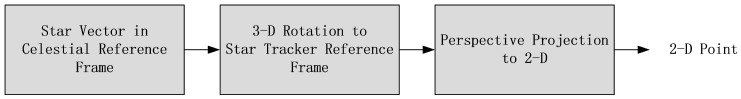
Measurement model of EKF.

The measurement noise v is zero mean, Gaussian sequence, with covariance matrix R. Measurement function $h_{k}$ comprises the 3-D rotation and perspective projection functions shown in Figure 2.

The rotation matrix which describes the rotation from celestial reference frame to star sensor reference frame could be defined in terms of a unit quaternion [14]:

(13) $$R=\left[\begin{array}{ccc} q_{0}^{2}+q_{1}^{2}-q_{2}^{2}-q_{3}^{2} & 2(q_{1}q_{2}+q_{0}q_{3}) & 2(q_{1}q_{3}-q_{0}q_{2})\\ 2(q_{1}q_{2}-q_{0}q_{3}) & q_{0}^{2}-q_{1}^{2}+q_{2}^{2}-q_{3}^{2} & 2(q_{2}q_{3}+q_{0}q_{1})\\ 2(q_{1}q_{3}+q_{0}q_{2}) & 2(q_{2}q_{3}-q_{0}q_{1}) & q_{0}^{2}-q_{1}^{2}-q_{2}^{2}+q_{3}^{2} \end{array}\right]$$

Let $r=(r_{x},r_{y},r_{z})$ be the 3-D coordinate of a unit vector in star sensor reference frame and $s=(s_{x},s_{y},s_{z})$ in celestial reference frame:

(14) r=Rs

The star locations $\left(x_{i},y_{i}\right)$ is then calculated by using:

(15) $$x_{i}=-f\frac{r_{x}}{r_{z}},y_{i}=-f\frac{r_{y}}{r_{z}}$$

where *f* is the camera focal length.

The linearized measurement matrix is the partial derivative of the measurement function with respect to each state variable and evaluated at time *t = t_k_*. Formally, **H***_k_* is determined as:

(16) $$H_{k}\left(s_{k}\right)={\frac{\partial h\left(s\right)}{\partial s}|}_{s=s_{k}}=\left(\left(\begin{array}{ccc} \frac{\partial x_{1}}{\partial s_{1}} & \cdots & \frac{\partial x_{1}}{\partial s_{7}}\\ \vdots & \ddots & \vdots \\ \frac{\partial y_{n}}{\partial s_{1}} & \cdots & \frac{\partial y_{n}}{\partial s_{7}} \end{array}\right)\right)$$

**H***_k_* is a 2n-by-7 matrix where n is the number of matched stars. For each matched star, the partial derivative of each coordinate needs to be computed:

(17) $$\begin{array}{l} \frac{\partial x}{\partial s_{i}}=\frac{\partial x}{\partial r_{x}}\frac{\partial r_{x}}{\partial s_{i}}+\frac{\partial x}{\partial r_{y}}\frac{\partial r_{y}}{\partial s_{i}}+\frac{\partial x}{\partial r_{z}}\frac{\partial r_{z}}{\partial s_{i}}\\ \frac{\partial y}{\partial s_{i}}=\frac{\partial y}{\partial r_{x}}\frac{\partial r_{x}}{\partial s_{i}}+\frac{\partial y}{\partial r_{y}}\frac{\partial r_{y}}{\partial s_{i}}+\frac{\partial y}{\partial r_{z}}\frac{\partial r_{z}}{\partial s_{i}} \end{array}$$

The partial derivatives of the observed stars x and y with respect to each element of **r** are given below:

(18) $$\begin{array}{l} \frac{\partial x}{\partial r_{x}}=-\frac{f_{c}}{r_{z}},\frac{\partial x}{\partial r_{y}}=0,\frac{\partial x}{\partial r_{z}}=\frac{r_{x}f_{c}}{r_{z}^{2}}\\ \frac{\partial y}{\partial r_{x}}=0,\frac{\partial y}{\partial r_{y}}=-\frac{f_{c}}{r_{z}},\frac{\partial y}{\partial r_{z}}=\frac{r_{y}f_{c}}{r_{z}^{2}} \end{array}$$

The partial derivatives of **r** with respect to each state variable can be calculated:

(19) $$\frac{\partial r}{\partial w_{i}}=0\left(i=x,y,z\right),\frac{\partial r}{\partial q_{i}}=\left(\begin{array}{c} \frac{\partial r_{x}}{\partial q_{i}}\\ \frac{\partial r_{y}}{\partial q_{i}}\\ \frac{\partial r_{z}}{\partial q_{i}} \end{array}\right)=\frac{\partial R}{\partial q_{i}}s(i=0,1,2,3)$$

The partial derivatives of **R** with respect to each rotational quaternion variable are:

(20) $$\begin{array}{l} \frac{\partial R}{\partial q_{0}}=\left[\begin{array}{ccc} 2q_{0} & 2q_{3} & -2q_{2}\\ -2q_{3} & 2q_{0} & 2q_{1}\\ 2q_{2} & -2q_{1} & 2q_{0} \end{array}\right],\frac{\partial R}{\partial q_{1}}=\left[\begin{array}{ccc} 2q_{1} & 2q_{2} & 2q_{3}\\ 2q_{2} & -2q_{1} & 2q_{0}\\ 2q_{3} & -2q_{0} & -2q_{1} \end{array}\right]\\ \frac{\partial R}{\partial q_{2}}=\left[\begin{array}{ccc} -2q_{2} & 2q_{1} & -2q_{0}\\ 2q_{1} & 2q_{2} & 2q_{3}\\ 2q_{0} & 2q_{3} & -2q_{2} \end{array}\right],\frac{\partial R}{\partial q_{3}}=\left[\begin{array}{ccc} -2q_{3} & 2q_{0} & 2q_{1}\\ -2q_{0} & -2q_{3} & 2q_{2}\\ 2q_{1} & 2q_{2} & 2q_{3} \end{array}\right] \end{array}$$

**Figure 3:**
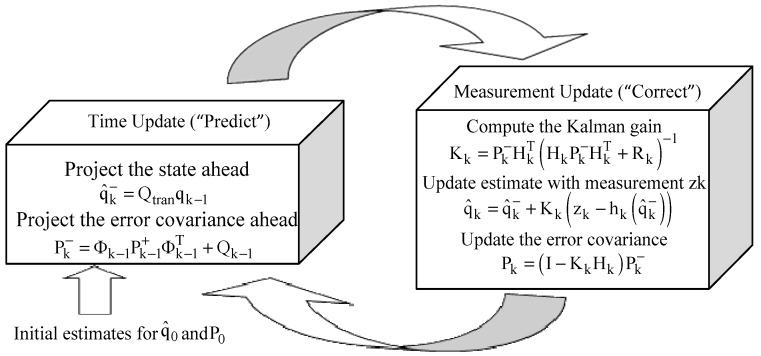
Operation of the EKF.

Figure 3 below offers a complete picture of the operation of the EKF, The time update projects the current state estimate ahead in time. The measurement update adjusts the projected estimate by an actual measurement at that time. The elements of the EKF are reported in Table 1. The (−) and (+) notations represent the estimate before and after the measurement update, respectively.

### 2.3. Star Catalog Partition

**Figure 4:**
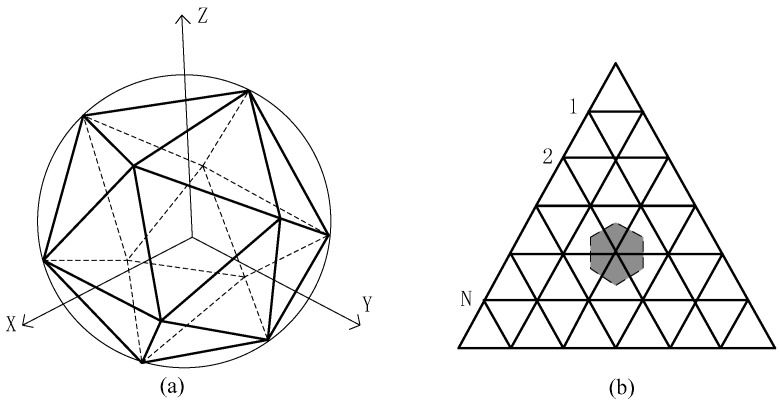
Star catalog partition model. (**a**) The celestial sphere is divided into twenty equivalent zones by an inscribed icosahedron. (**b**) Each of twenty regions is divided into several congruent equilateral triangles.

In order to speed up accessing the cataloged stars to identify the stars which enter or exit the sensor FOV, the celestial sky is divided nearly evenly spaced and completely covering of the sphere. Ideally, the vertices of a regular polyhedron represent evenly spaced points on a sphere. Unfortunately there are a limited number of regular polyhedrons, none of which provide enough points. However, the faces of the polyhedron can be divided into equal polygons with the vertices projected on the sphere to get enough points. Clearly, the minimal nonuniformity will result from the polyhedron with the most and consequently smallest faces. Also, the triangular or square faces would be the simplest to subdivide. The icosahedron, a regular 20-sided polygon whose faces are equilateral triangles, fits both requirements. The partitioning steps are as follows:

① The celestial sphere can be divided into twenty equivalent zones by an inscribed icosahedron which is shown in Figure 4a. A pyramid is formed by connecting the center of the celestial sphere with the three vertices on one face of the icosahedron. The pyramids divide the celestial sphere totally into twenty equivalent zones.
② Each of twenty regions is divided into several congruent equilateral triangles. As shown in the Figure 4b, each edge is divided into N + 1 segments, and each region is divided into ${\left(N+1\right)}^{2}$ triangles.
③ When projected on the sphere, the vertices of these smaller triangles become the $n=10N^{2}+20N+12$ centers of sub-catalogs that completely cover the sphere, called sub-catalog centers. Each star in the catalog is then assigned to the sub-catalog of closest vertex. The sub-catalog centers number is computed as follows:
  (21) n=12+30×N+20×N×(N−1)/2

The element of the partition table is structured as Table 2, which is arranged in descending order of the x component of the center vector.

**Figure 5:**
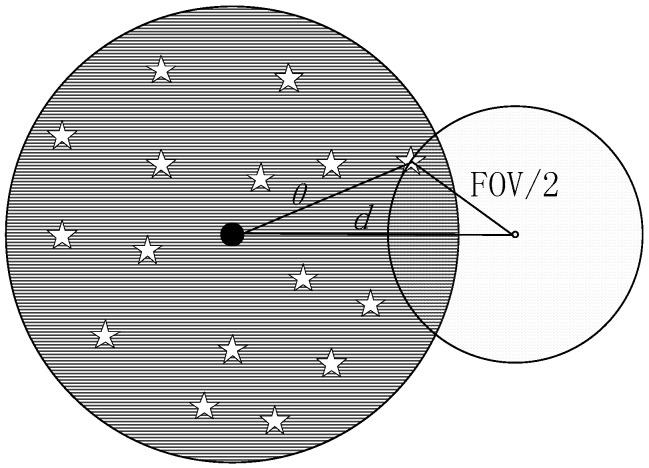
Necessary condition for stars to enter the FOV. The dark gray area represents sub-catalog, and the light gray area represents FOV.

If the boresight of a star sensor is roughly known, the subcatalogs around the boresight are to be found quickly, so the guide stars within the FOV can be accessed immediately. The term *θ* is used to represent the maximum angle between the guide stars and the center of subcatalog. As shown in Figure 5, the necessary condition for stars to enter the FOV is that the angle between the center of the subcatalog and the boresight of the star sensor denoted by *d* is less than *θ* + FOV/2.

Let the angle between the boresight and the OXY plane of celestial reference frame be $\alpha_{z}$, then the lower and upper bound of z component of the center vector are respectively:

(22a) $$\begin{array}{l} z_{l}=\sin\left(\max\left\{\alpha_{z}-\left(\theta +\frac{\mathrm{FOV}}{2}\right),-\pi /2\right\}\right)\\ z_{u}=\sin\left(\min\left\{\alpha_{z}+\left(\theta +\frac{\mathrm{FOV}}{2}\right),\pi /2\right\}\right) \end{array}$$

Similarly, let the angle between the boresight and the OYZ plane be $\alpha_{x}$, and the angle between the boresight and the OZX plane be $\alpha_{y}$, then the lower and upper bound of x and y component of the center vector are respectively:

(22b) $$\begin{array}{l} x_{l}=\sin\left(\max\left\{\alpha_{x}-\left(\theta +\frac{\mathrm{FOV}}{2}\right),-\pi /2\right\}\right)\\ x_{u}=\sin\left(\min\left\{\alpha_{x}+\left(\theta +\frac{\mathrm{FOV}}{2}\right),\pi /2\right\}\right)\\ y_{l}=\sin\left(\max\left\{\alpha_{y}-\left(\theta +\frac{\mathrm{FOV}}{2}\right),-\pi /2\right\}\right)\\ y_{u}=\sin\left(\min\left\{\alpha_{y}+\left(\theta +\frac{\mathrm{FOV}}{2}\right),\pi /2\right\}\right) \end{array}$$

The necessary condition for stars to enter the FOV can be expressed as Equation (23):

(23) $$\begin{array}{l} x_{l}<x<x_{u}\\ y_{l}<y<y_{u}\\ z_{l}<z<z_{u} \end{array}$$

Hence, the search of subcatalogs around the boresight is accomplished using spherical polygon approach [1] and standard Binary Search technique. The above star catalog partition method greatly reduces searching time, while needs less memory space comparing the methods in ref. [1,4], as shown in Section 3.

### 2.4. Initial Angular Velocity Estimation

Initial angular velocity is derived from the quaternion difference. Taking account of Equation (5), the quaternion difference is written as:

(24) $$\Delta q\left(t\right)=\left[\begin{array}{c} \begin{array}{c} \Delta q_{0}\left(t\right)\\ \Delta q_{1}\left(t\right) \end{array}\\ \begin{array}{c} \Delta q_{2}\left(t\right)\\ \Delta q_{3}\left(t\right) \end{array} \end{array}\right]\equiv q\left(t_{2}\right)⊗q^{*}\left(t_{1}\right)=\left[\begin{array}{c} \cos\left(\frac{\left\|\Omega \right\|\left(t_{2}-t_{1}\right)}{2}\right)\\ \frac{w_{x}}{\left\|\Omega \right\|}\sin\left(\frac{\left\|\Omega \right\|\left(t_{2}-t_{1}\right)}{2}\right)\\ \frac{w_{y}}{\left\|\Omega \right\|}\sin\left(\frac{\left\|\Omega \right\|\left(t_{2}-t_{1}\right)}{2}\right)\\ \frac{w_{z}}{\left\|\Omega \right\|}\sin\left(\frac{\left\|\Omega \right\|\left(t_{2}-t_{1}\right)}{2}\right) \end{array}\right]$$

Taylor series expansion of $\Delta q_{1}\left(t\right)$ is:

$$\Delta q_{1}\left(t\right)=\frac{w_{x}\left(t_{2}-t_{1}\right)}{2}-\frac{w_{x}{\left\|\Omega \right\|}^{2}{\left(t_{2}-t_{1}\right)}^{3}}{48}+0\left({\left\|\Omega \right\|}^{4}\right)\approx \frac{w_{x}\left(t_{2}-t_{1}\right)}{2}$$

The linear term is used to approximate $w_{x}$:

(25a) $$w_{x}\approx \Delta q_{1}\left(t\right)\times \frac{2}{t_{2}-t_{1}}$$

Similarly, we have:

(25b) $$w_{y}\approx \Delta q_{2}\left(t\right)\times \frac{2}{t_{2}-t_{1}},w_{z}\approx \Delta q_{3}\left(t\right)\times \frac{2}{t_{2}-t_{1}}$$

Typically, a star sensor slews within 8°/s [15,16,17,18] and operates with an update rate of 10 Hz inducing some error less than 1 arcsec/s, so this approximation is reasonable.

Equation (25) can be expanded as:

(26) $$w=\frac{2}{t_{k+1}-t_{k}}\left[\begin{array}{cccc} \begin{array}{c} q_{1}(k+1)\\ q_{2}(k+1)\\ q_{3}(k+1) \end{array} & \begin{array}{c} q_{0}(k+1)\\ q_{3}(k+1)\\ -q_{2}(k+1) \end{array} & \begin{array}{c} -q_{3}(k+1)\\ q_{0}(k+1)\\ q_{1}(k+1) \end{array} & \begin{array}{c} q_{2}(k+1)\\ -q_{1}(k+1)\\ q_{0}(k+1) \end{array} \end{array}\right]\left[\begin{array}{c} q_{0}(k)\\ -q_{1}(k)\\ -q_{2}(k)\\ -q_{3}(k) \end{array}\right]=\frac{2}{t_{k+1}-t_{k}}\left[\begin{array}{cccc} \begin{array}{c} -q_{1}(k)\\ -q_{2}(k)\\ -q_{3}(k) \end{array} & \begin{array}{c} q_{0}(k)\\ q_{3}(k)\\ -q_{2}(k) \end{array} & \begin{array}{c} -q_{3}(k)\\ q_{0}(k)\\ q_{1}(k) \end{array} & \begin{array}{c} q_{2}(k)\\ -q_{1}(k)\\ q_{0}(k) \end{array} \end{array}\right]\left[\begin{array}{c} q_{0}(k+1)\\ q_{1}(k+1)\\ q_{2}(k+1)\\ q_{3}(k+1) \end{array}\right]$$

### 2.5. Track Window Radius Determination

In the tracking mode, the stars are measured and evaluated in the star image tracking windows. If there is only one observed star in a window, the star is matched with the reference star corresponding to the track window. The larger the radius, the greater the probability that the window contains false stars due to hot spots, space radiation, stray light, etc. In order to improve tracking reliability, the tracking window radius should be as small as possible. The radius of the window is determined by the accuracy of the predicted attitude. It is possible to estimate the average star prediction accuracy, $E_{\mathrm{boresight}}$, by Equation (27):

(27) $$E_{\mathrm{prediction}}=\frac{E_{\mathrm{boresight}}\times N_{\mathrm{pixel}}\times \sqrt{N_{\mathrm{star}}}}{FOV}$$

where $E_{\mathrm{boresight}}$ is the predicted boresight accuracy obtained from the EKF error covariance extrapolation, $N_{\mathrm{pixel}}$ is the number of pixels across the focal plane, and $N_{\mathrm{star}}$ is the number of stars detected on the camera image.

In order to improve the location accuracy of the star, the spot size of the star image is enlarged to a few pixels ranging from 3 to 5. Hence, the tracking window radius could be determined by Equation (28):

(28) $$R_{\mathrm{track}}=5\times E_{\mathrm{prediction}}+5$$

## 3. Simulation and Experiment

Several simulations and night sky tests were performed to evaluate the tracking algorithm presented in this paper. The simulations were implemented in MATLAB in the Microsoft Windows environment on a Core II 2.8 GHz PC.

### 3.1. Simulation

The star sensor configuration used for the simulations reported in this paper uses a 14.5 degrees circular FOV and 44.27 mm focal distance with a focal plane consisting of 2048 × 2048 pixels. A guide star catalog is made of 4338 stars below magnitude 6, based on the Smithsonian Astrophysical Observatory (SAO) J2000 star catalogue. From the discussion below parameter N in the star catalog partition method is 4, so the number of sub-catalogs divided is 252.

Simulation has been performed to evaluate the performance of the attitude tracking method under a variety of conditions. The results are obtained by varying initial conditions to characterize errors, convergence, and stability in the attitude quaternion estimate. Root mean square errors are presented.

Initial conditions requiring specification include the initial attitude and the error covariance matrix which are obtained from the iterative identification algorithm [19] and the QUEST algorithm [5]. Process noise given by the covariance matrix Q and measurement noise given by the covariance matrix R is also specified initially, remaining constant throughout. Table 3 gives the initial states for the simulation, as well as the assumed initial error covariance diagonal terms and the process noise covariance diagonal terms.

Actual initial attitude quaternion is (0.54435, 0.15051, −0.07010, 0.82226), and true angular rate is (−0.03, 0.04, −0.02) rad/s. The angular velocity is assumed to be constant in the tracking models. Taking into account the actual conditions, the fluctuation in angular velocity is assumed to be 0.001 rad per second. Hence, the process noise covariance diagonal terms are {1 × 10^−^^9^, 1 × 10^−^^9^, 1 × 10^−^^9^, 1 × 10^−^^9^, 1 × 10^−6^, 1 × 10^−6^, 1 × 10^−6^}, while the off-diagonal terms are zero. The star measurement accuracy is determined by the star signal-to-noise ratio (SNR). The higher the SNR is, the more accuracy a star sensor can achieve. The measurement noise is a Gaussian with a standard deviation ranging from 0.04 to 0.18 pixel [18]. Since the noise for the points is independent with respect to x and y as well as each other, the measurement error covariance matrix is a straightforward diagonal matrix whose diagonal elements are the noise variances while the off-diagonal terms are zero. The simulated sample interval is 0.1 s.

**Figure 6:**
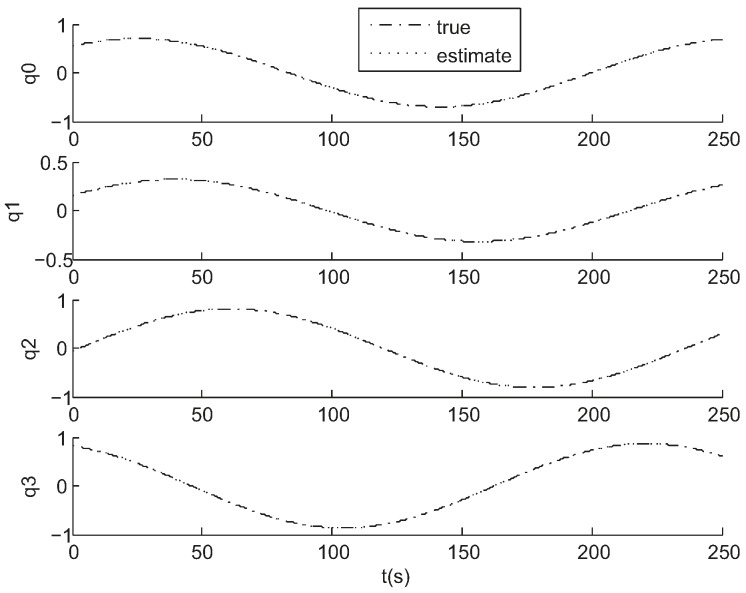
Attitude estimation results in simulation.

**Figure 7:**
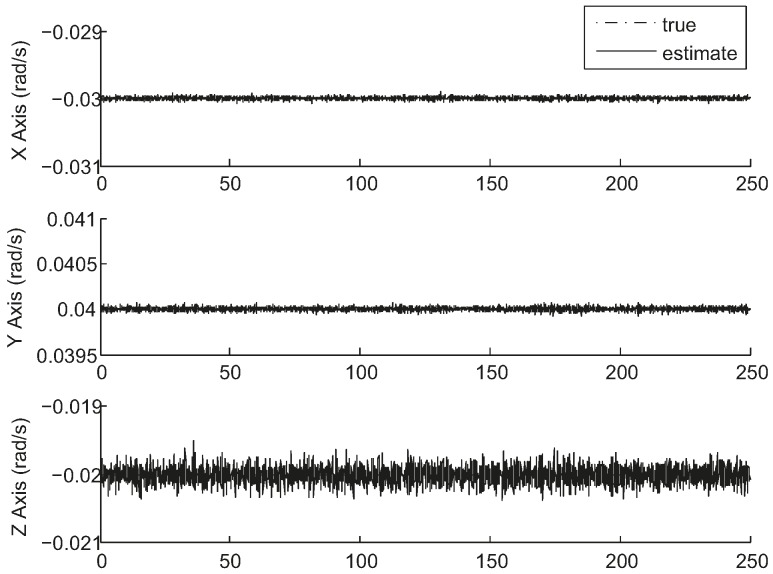
Angular velocity estimation results in simulation.

Figure 6 and Figure 7 show the results of a sample run where the true states are shown along with the estimated values. The simulation was run for a time of 250 s with a measurement interval of 0.1 s. The tracking is always successful.

**Figure 8:**
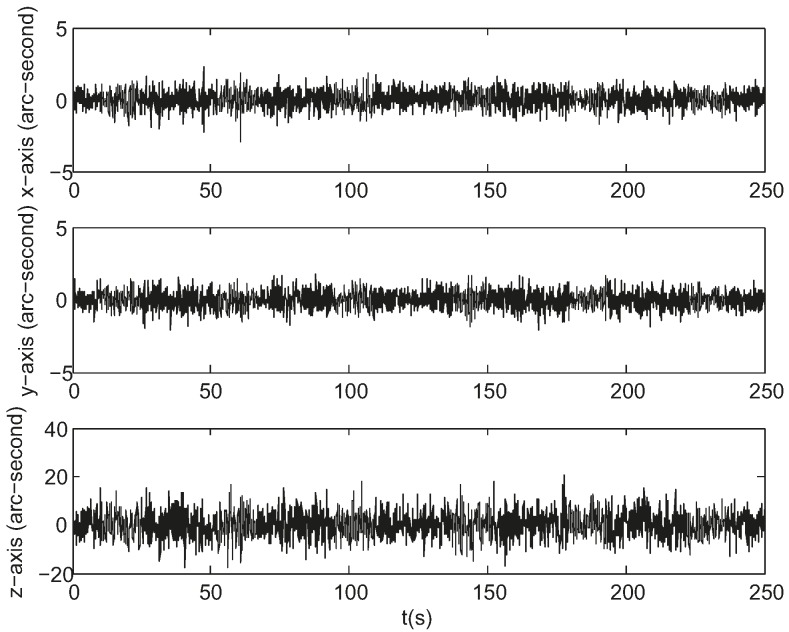
Attitude estimation errors in simulation.

**Figure 9:**
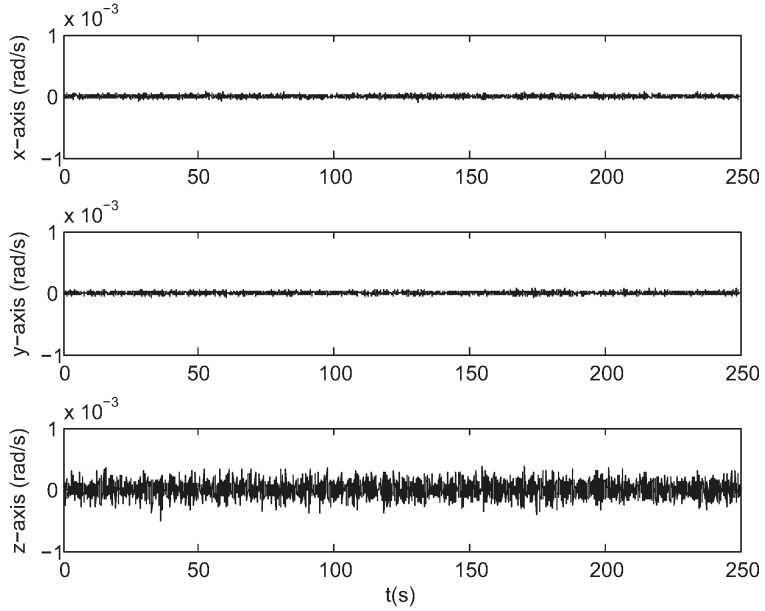
Angular velocity estimation errors in simulation.

Figure 8 gives the attitude estimation errors over the same period of time from the same sample data represented in Euler angles around the *x*-, *y*- and *z*-axis, whose standard deviations are 0.5, 0.5, and 5.7 arc seconds respectively. Figure 9 gives the estimation errors for angular velocity about the *x*-, *y*- and *z*-axis whose standard deviations are 2.6 × 10^−^^5^, 2.3 × 10^−^^5^, 1.3 × 10^−^^4^ rad/s respectively. The accuracy along the boresight of the star sensor (*z*-axis) is less than the accuracy cross the boresight (*x*- and *y*-axis), which is an inherent characteristic of a star sensor. The ratio of the uncertainty along the boresight to the uncertainty cross the boresight spans from 7 to 90 [20]. As can be seen, the estimates track the state variables reasonably well.

**Figure 10:**
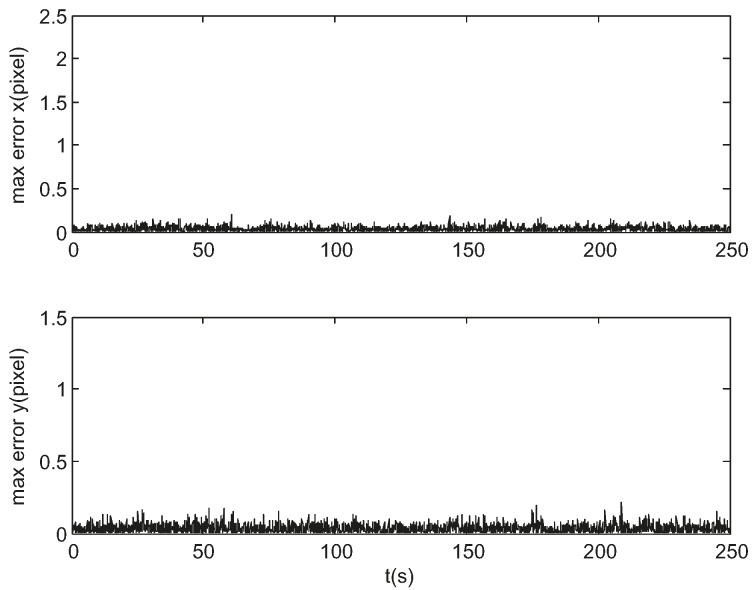
Maximum prediction error of the star locations in simulation.

Figure 10 shows the maximum prediction error of the star locations in each tracking cycle. It is noted that the initial angular velocity estimations are inaccurate so that the prediction errors in the first frame are larger which are 2.27, 1.45 pixels respectively about x- and y-coordinate. The prediction errors in the next frame are less than 0.2 pixel.

**Figure 11:**
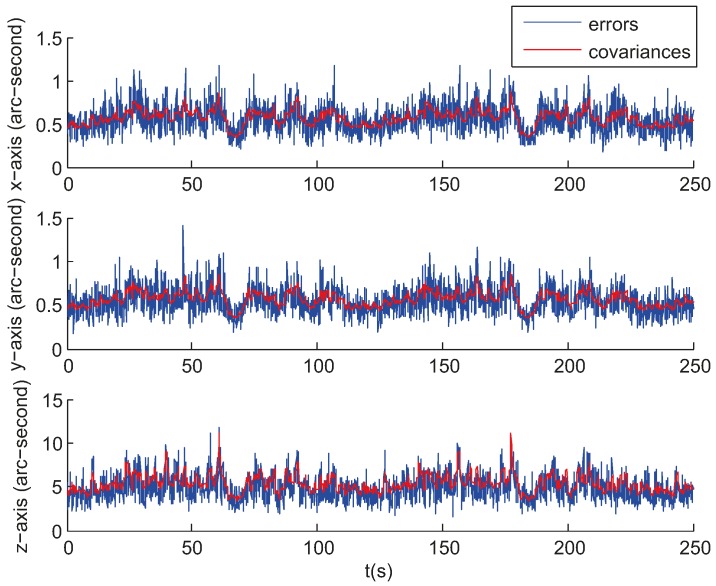
Root mean square estimation error and square root of the error covariance from the Kalman filter for attitude estimation.

**Figure 12:**
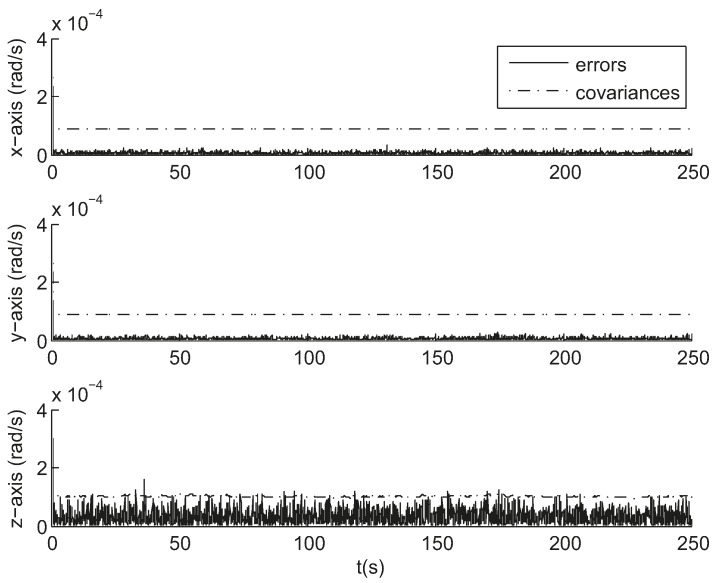
Root mean square estimation error and square root of the error covariance from the Kalman filter for angular velocity estimation.

The smaller the error is, the smaller the size of track windows is, and the more robust the tracking is. As the star location prediction error is relatively small, as shown in Figure 10, the radius of the track windows is set to 16 pixels ensuring a unique match and not updated according to the EKF error covariance. The above results are for a single representative run and are not necessarily typical. A further measure of performance is the error over a large number of trials. Using the same initial conditions given above, a series of tests were performed to experimentally measure the error. Figure 11 and Figure 12 show the calculated root mean square (RMS) error over 100 sample runs. The square root of the corresponding mean diagonal element from the calculated covariance error matrix is also shown in each figure. In the nonlinear EKF, the covariance matrix depends on the measurements and does not necessarily represent the actual error covariance [21]. In these tests, the covariance matrix values correspond closely to the actual error values for the attitude estimation while minor differences are present for the angular velocity estimation.

### 3.2. Night Sky Experiments

**Figure 13:**
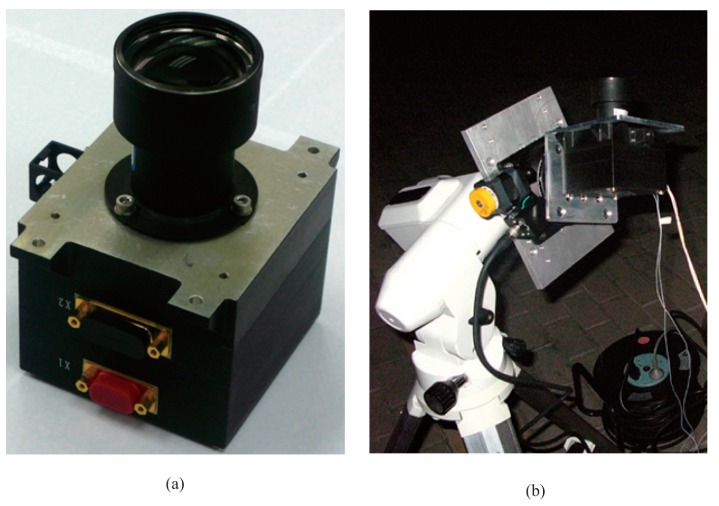
Star sensor used for the night sky experiments. (**a**) Our own star sensor. (**b**) Night sky experiments.

The night sky tests have been performed at the Xinglong Astronomical Observatory of the Chinese Academy of Sciences. The star sensor was placed on a rotating table with vertical axis as shown in Figure 13. The parameters of the star sensor are listed in Table 4.

**Figure 14:**
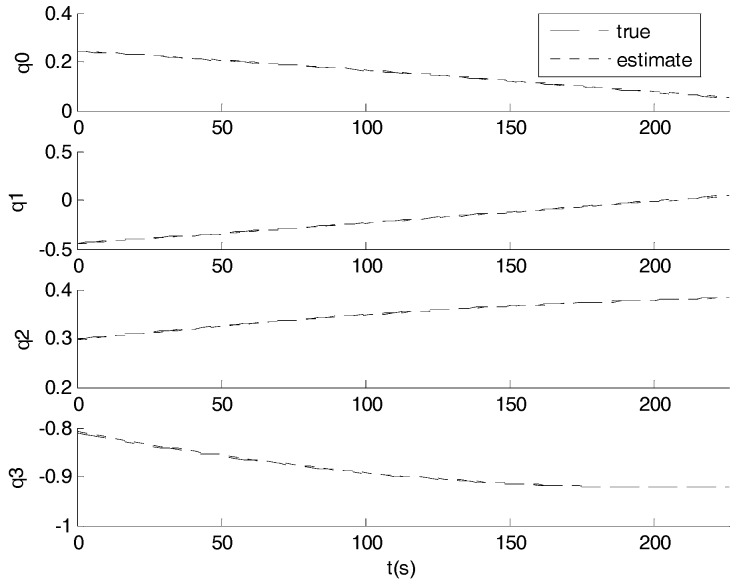
Attitude estimation results of a night sky experiment.

Limited by the sensitivity of the image sensor, the angular rate is set to 2 degrees per second around *y*-axis of the star sensor. The estimated quaternion is plotted in Figure 14.

**Figure 15:**
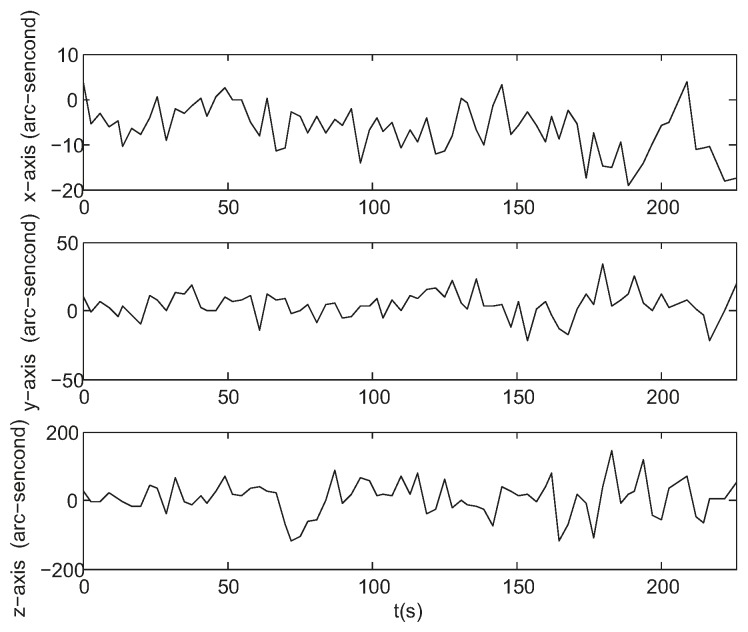
Attitude estimation errors of a night sky experiment.

**Figure 16:**
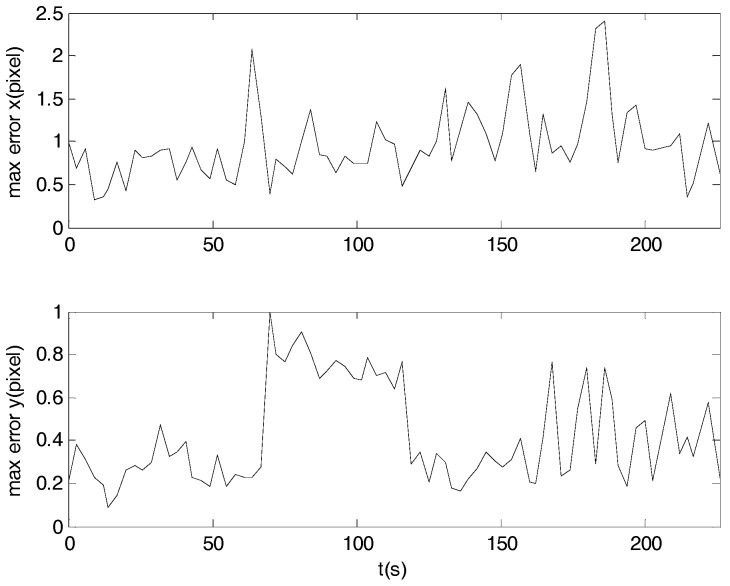
Maximum star location prediction error of a night sky experiment.

In order to assess the 3-axis attitude measurement accuracy we create a reference quaternion (qr) which represents as good as possible the trajectory track under investigation. For that purpose the four elements of the measured quaternions were fitted over their time stamp to a 5th degree polynomial. The difference between the reference quaternion which is smooth due to 5th order polynomial fitting and the measured quaternion both at the same time stamp represents the attitude error which can be expressed as Euler angle rotation around *x*-, *y*-, *z*-axis as shown in Figure 15. The standard deviations are 5.3, 9.8, and 50.8 arc seconds respectively, which are larger than simulation errors due to the larger star position error caused by low SNR value, the less star number detected in the field of view caused by low star sensitivity and the more star vector uncertainty caused by atmosphere turbulence. It can be seen that the *y*-axis error is larger than the *x*-axis error due to introduction of system error of rotating table. It is shown in Figure 16 that the prediction error of the star locations is less than 3 pixels. 

**Figure 17:**
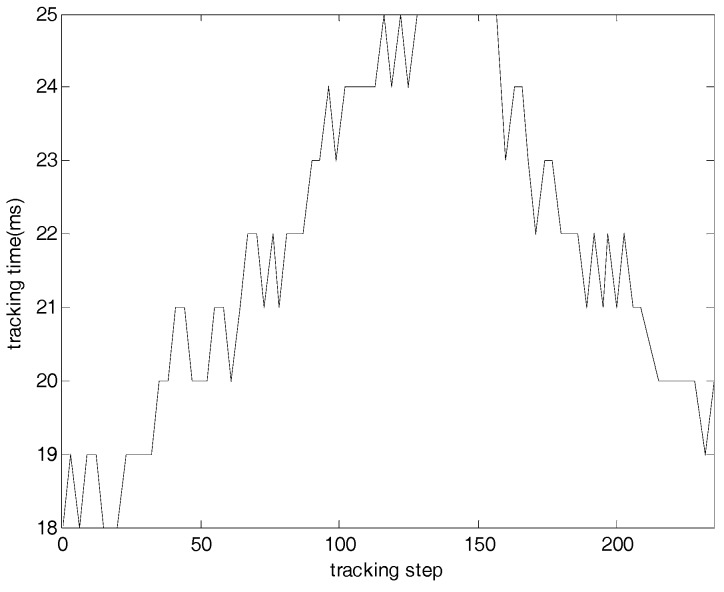
Tracking time for a real star sensor.

The tracking time in every cycle is shown in Figure 17. The tracking time in every cycle keeps less than 40 milliseconds, so this algorithm is suitable for real-time tracking.

### 3.3. Comparison and Analysis

**Figure 18:**
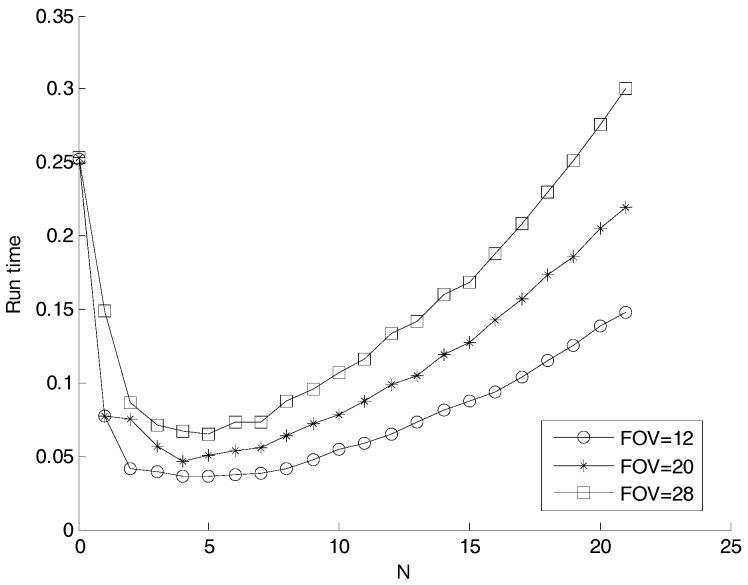
Time to simulate the reference star image versus N.

The time to predict the frame of the stars at each time step constituted with the time to search the partition table and the time to access the catalog is related with the number of subcatalogs. The larger the subcatalog number is, the longer the time searching the partition table is. However, the average number of the stars in one subcatloag decreases as the subcatalog number increases, and it will take shorter time to access the catalog. To determine the optimum number of subcatalogs, the distribution of the stars on the celestial sphere was assumed to be uniform, and the time to simulate the reference star image was computed against N using a Monte Carlo simulation method. The time is normalization to that accessing all stars in the catalog. The result is shown in Figure 18. For an FOV of Φ20°, the time consumed is 0.08 when N is 11. According to the results, to achieve the shortest consumed time, N is decided to be in the range of 3 to 5.

**Figure 19:**
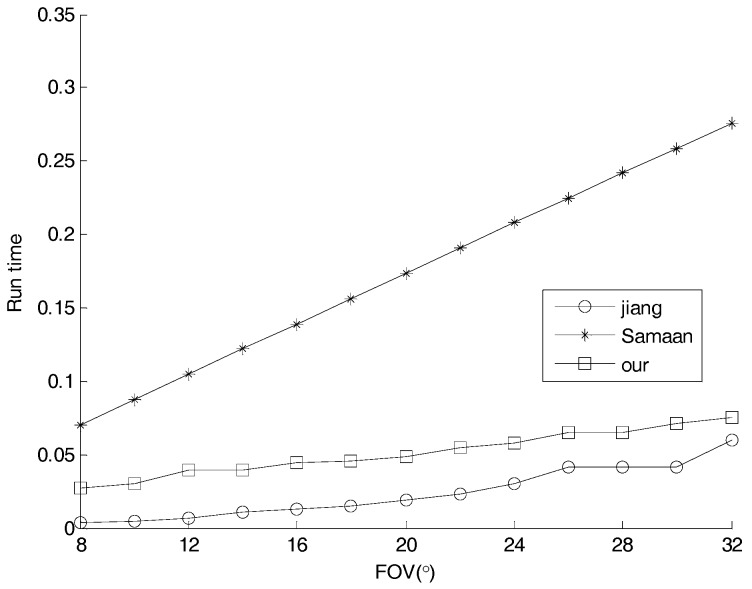
Time to simulate the reference star image versus FOV.

To compare the speed of Samaan [1] and Jiang [4], the time to simulate the reference star image was computed against FOV by Monte Carlo simulation. The result is show in Figure 19. The time taken by our method is shorter than ref. [1], but longer than ref. [4]. As FOV increases, the gap between ours and [4] is reduced, and that between ours and [1] is enlarged. For an FOV of Φ20°, the time consumed is 0.05, decreased by 70 percent compared to [1] and increased by 50 percent more than [4].

A comparison in consumed memory among these methods is listed below. For an FOV of Φ20° and catalog star number of 5000, our methods needs 5 k bytes, which is a decrease by 83 percent compared to [4] and by 91 percent compared to [1].

## 4. Conclusions

To improve the performance of star sensor in case of high attitude dynamics, a novel attitude tracking algorithm based on extended Kalman filter is presented. The approach model the star sensor as a nonlinear stochastic system with the state estimate providing the three degree-of-freedom attitude quaternion and angular velocity based on the extended Kalman filter (EKF). From EKF the attitude quaternion and its error covariance are extrapolated to the next frame used to predict the centers and radius of the star track windows. In order to speed up the prediction a star catalogue partition method by an inscribed regular icosahedron is introduced for a fast search of the star catalog while having a small memory requirement. Then the matched stars in the track windows of star image are measured and used as the measurements for EKF to estimate the optimal attitude quaternion of current frame and the angular velocity.

The simulation and night sky experiment results demonstrate that this algorithm provides effective and reliable attitude tracking. In the simulation the EKF always converge, and the covariance matrix values accord with the actual error values for the attitude estimation while minor differences are present for the angular velocity estimation. In addition, it is noted that the accuracy along the boresight of the star sensor (*z*-axis) is less than the accuracy cross the boresight (*x*- and *y*-axis), which is an inherent characteristic of a star sensor. Moreover, it can be seen that the prediction error of the star locations is less than 3 pixels and the tracking time in every cycle keeps less than 40 milliseconds in the night sky experiment. Hence, the algorithm is particularly suitable for use in orbit as it is computationally efficient and has a small memory requirement. Future work can involve on-orbit validation of the attitude tracking algorithm.

## Figures and Tables

**Table 1:** EKF elements and descriptions.

Parameter	Description
ϕ	The state transition function
Φ	The linear term of the Taylor series expansion of the state transition function
Q	The process noise covariance matrix
P	The state error covariance matrix
K	The extended Kalman filter gain
h	The measurement function
H	The linear term of the Taylor series expansion of the measurement function
R	The measurement noise covariance matrix

**Table 2:** Structure of the element in the partition table.

Element Index	Subcatalog Center Direction	Subcatalog Start Address in Guide Catalog	Subcatalog Length	Maximum Angle between the Guide Stars in the Subcatalog and the Center (Degree)
1	(−0.99244712, 0.06449312, 0.10435207)	1	17	8.00
2	(−0.99244712, −0.06449312, −0.10435207)	18	9	6.37
…	…	…	…	…
n	(0.99244712, −0.06449312, −0.10435207)	4324	15	7.46

**Table 3:** Initial conditions for the simulation.

	Quaternion	Angular Velocity
True Initial State	(0.54680, 0.15199, −0.06565, 0.82073)	(−0.03, 0.04, −0.02) rad/s
Initial State Estimate	(0.54679, 0.15199, −0.06566, 0.82074)	(−0.03115, 0.04046, −0.02086) rad/s
Error Covariance	(6 × 10^−7^, 1 × 10^−8^, 3 × 10^−8^, 3 × 10^−7^)	(8 × 10^−4^, 8 × 10^−4^, 8 × 10^−4^) (rad/s)^2^
Process Noise	(1 × 10^−9^, 1 × 10^−9^, 1 × 10^−9^, 1 × 10^−9^)	(1 × 10^−6^, 1 × 10^−6^, 1 × 10^−6^) (rad/s)^2^

**Table 4:** Star sensor parameters for the night sky test.

Parameter	Value
Initial attitude	(0.24383, −0.44028, 0.29841, −0.81095)
Angular velocity	(0, 0.03491, 0) rad/s
Focal length	44.27 mm
FOV	14.5 degrees
Pixel array	2048 × 2048
